# Apolipoprotein(a) production and clearance are associated with plasma IL-6 and IL-18 levels, dependent on ethnicity

**DOI:** 10.1016/j.atherosclerosis.2024.117474

**Published:** 2024-02-13

**Authors:** Anouk G. Groenen, Anastasiya Matveyenko, Nelsa Matienzo, Benedek Halmos, Hanrui Zhang, Marit Westerterp, Gissette Reyes-Soffer

**Affiliations:** a Department of Pediatrics, University Medical Center Groningen, University of Groningen, Groningen, the Netherlands; b Columbia University Irving Medical Center, College of Physicians and Surgeons, Department of Medicine, Division of Preventive Medicine and Nutrition, New York, NY, USA; c Columbia University Irving Medical Center, Division of Cardiology, New York, NY, USA

**Keywords:** Lipoprotein(a), APO(a) kinetics, APO(a) isoform size, Inflammation, ASCVD

## Abstract

**Background and aims:**

High plasma lipoprotein (a) [Lp(a)] levels are associated with increased atherosclerotic cardiovascular disease (ASCVD), in part attributed to elevated inflammation. High plasma Lp(a) levels inversely correlate with apolipoprotein (a) [(APO(a)] isoform size. APO(a) isoform size is negatively associated with APO (a) production rate (PR) and positively associated with APO(a) fractional catabolic rate (FCR). We asked whether APO(a) PR and FCR (kinetics) are associated with plasma levels of interleukin (IL)-6 and IL-18, pro-inflammatory interleukins that promote ASCVD.

**Methods:**

We used samples from existing data of APO(a) kinetic studies from an ethnically diverse cohort (n = 25: 10 Black, 9 Hispanic, and 6 White subjects) and assessed IL-6 and IL-18 plasma levels. We performed multivariate linear regression analyses to examine the relationships between predictors APO(a) PR or APO(a) FCR, and outcome variables IL-6 or IL-18. In these analyses, we adjusted for parameters known to affect Lp(a) levels and APO(a) PR and FCR, including race/ethnicity and APO(a) isoform size.

**Results:**

APO(a) PR and FCR were positively associated with plasma IL-6, independent of isoform size, and dependent on race/ethnicity. APO(a) PR was positively associated with plasma IL-18, independent of isoform size and race/ethnicity. APO(a) FCR was not associated with plasma IL-18.

**Conclusions:**

Our studies demonstrate a relationship between APO(a) PR and FCR and plasma IL-6 or IL-18, interleukins that promote ASCVD. These studies provide new insights into Lp(a) pro-inflammatory properties and are especially relevant in view of therapies targeting APO(a) to decrease cardiovascular risk.

## 1. Introduction

Genome wide association studies, epidemiological, and clinical studies have established high plasma lipoprotein(a) [Lp(a)] as a causal risk factor for atherosclerotic cardiovascular disease (ASCVD) [1–4]. High plasma Lp(a) levels are associated with atherosclerosis, thrombosis, and arterial calcification [5]. Various Lp(a) lowering therapies are in phase 2 and 3 development [6–11]. Therefore, there is an urgency to understand the relationship between Lp(a) levels and disease presentation. Lp(a) contains two main apolipoproteins: apolipoprotein(a) [APO (a)] and apolipoprotein B100 [APOB100]. Plasma Lp(a) levels are largely controlled by the *LPA* gene, which encodes APO(a). The Kringle IV type 2 (KIV_2_) domain of APO(a) ranges from 1 to >40 identical copies, reflecting heterogeneity in APO(a) isoform size. Plasma Lp(a) levels inversely correlate with APO(a) isoform size [1–4]. Smaller APO (a) isoforms correlate with high Lp(a) plasma levels and have high affinity for oxidized phospholipids (oxPL) [12–14]. OxPL carried by APO (a) and APOB100 components of Lp(a) [15] are key mediators of arterial wall inflammation [16], and have been associated with cardiovascular events [17–20].

Several *in vitro* studies have shown that Lp(a) enhances the expression of adhesion molecules in endothelial cells and pro-inflammatory interleukins in monocytes and macrophages [16,21–28], mainly driven by oxPL bound to Lp(a) [16,23,27]. Studies in humans have substantiated the relationship between Lp(a), inflammation, and ASCVD [16,20,29], as we recently reviewed [30]. While oxPL bound to Lp(a) clearly mediate Lp(a)’ pro-inflammatory effects [16,23,27], additional mechanisms may contribute.

Plasma Lp(a) levels are regulated by both APO(a) production rate (PR) and APO(a) fractional catabolic rate (FCR), *i.e.* APO(a) kinetics [31–35]. We here hypothesized, based on *in vitro* studies showing pro-inflammatory effects of Lp(a) [16,21–27], that APO(a) kinetics associate with plasma interleukins that promote future ASCVD. We hypothesize that APO(a) kinetics are a stronger predictor of inflammatory parameters than plasma Lp(a) levels itself, mainly because the small APO(a) isoform that is associated with ASCVD [36,37] is positively associated with APO(a) PR [31–35].

To test our hypothesis, we examined associations between APO(a) kinetics and interleukins that are known to promote future ASCVD in subjects without inflammatory diseases, no previous or current history of CVD and, otherwise healthy [38–40]. We focused on interleukin (IL)-6 and IL-18, because IL-6 receptor polymorphisms are linked with CVD in Mendelian randomization studies and meta-analyses [41,42]; the decrease in cardiovascular events in the *Canakinumab Anti-inflammatory Thrombosis Outcome Study* (CANTOS) was proportional to reductions in plasma IL-6 [43]; and the residual pro-inflammatory risk in CANTOS was attributed to plasma IL-6 and IL-18 [44]. We also included IL-18 binding protein (BP) that neutralizes IL-18 [45], and high sensitivity C reactive protein (hsCRP), a general marker of inflammation, in our analyses. Since recent work from our lab highlights the effects of self-reported race and ethnicity (SRRE) and APO (a) isoform size on APO(a) kinetics [35,46], we examined these associations in a multi-ethnic cohort [38–40] taking isoform size and SRRE into account.

In examining potential associations, we are aware of data showing that in specific inflammatory conditions, IL-6 may increase plasma Lp(a) levels [47,48]. Although plasma Lp(a) levels are mostly genetically determined [49,50], plasma Lp(a) levels are elevated in rheumatoid arthritis (RA), chronic kidney disease (CKD) and in early COVID-19, characterized by the cytokine storm [47,48,51]. Pharmaceutical interventions have demonstrated that in RA and CKD, these increases were IL-6 dependent [47,48], indicating a direct link between elevated IL-6 and Lp(a). However, Lp(a) levels are only elevated when plasma IL-6 levels reach a certain threshold in conditions associated with elevated inflammation [52]. Since the subjects we examined were healthy and had no inflammatory diseases, our *a priori* hypothesis was that APO(a) PR and APO(a) FCR associate with plasma IL-6 and IL-18. Nonetheless, in view of the studies in inflammatory conditions [47,48,51], and the APO(a) promoter containing IL-6 response elements [52,53], we also examined the reverse associations.

## 2. Patients and methods

### 2.1. Study population

Study subjects were enrolled from previously completed APO(a) kinetic studies [38–40]. The study protocol conforms to the ethical guidelines of the 1975 Declaration of Helsinki and was approved by the Columbia University Irving Medical Center (CUIMC) Institutional Review Board (IRB). All subjects signed an informed consent form before enrollment and agreed to use of their data and samples for future research. We used pre-intervention phase plasma samples and data from 26 individuals with varying SRRE (Black, Hispanic, and White). Our study sample size did not allow us to examine the effects of each race separately in any associations. We used 12 h fasting plasma samples (appropriately stored at −80 °C, and not previously thawed) and baseline characteristics data collected during the original studies. We examined relationships between APO(a) PR or APO(a) FCR and IL-6, IL-18, IL-18BP and hsCRP.

### 2.2. Plasma Lp(a), APO(a) isoform size, and measurements of oxidized phospholipids on APO(a) and APOB100

Plasma Lp(a) levels (nmol/L) were measured using a monoclonal antibody-based ELISA method by the Northwest Lipid Metabolism and Diabetes Research Laboratory [54]. This method is validated to accurately determine Lp(a) concentrations independently of KIV_2_ repeats of APO(a). Plasma APO(a) isoforms were estimated by a high-sensitive gel electrophoresis method [35,54,55]. In brief, 250 μL of plasma was diluted to 100 ng of protein in 40 μL using saline and combined with 40 μL reducing buffer. The sample was run on an agarose gel, transferred to a nitrocellulose membrane, immunoblotted, and imaged using the ChemiDoc MP Imaging System. Isoforms were determined by comparison to in-house standards of combined material containing six APO(a) isoforms: 38, 32, 24, 19, 15, and 12 KIV_2_ repeats. The relative expression of each isoform was determined based on the intensity profile of the two isoforms using Image Lab software. The method has an intra-sample variability under 15%.

A chemiluminescent sandwich ELISA was used to measure oxPL per unit of APO(a) captured in plasma [12,13]. Briefly, plasma was added to plates coated overnight with the monoclonal antibody LPA4 [56]. OxPLs were detected with biotinylated E06 in relative light units (RLU)/APO (a). Using the same ELISA, oxPL per unit of APOB100 was assessed [12, 13]; however, the Lp(a) was not immunoprecipitated prior to the assay. Therefore, we cannot distinguish whether oxPL was bound to APOB100 on LDL or Lp(a).

### 2.3. APO(a) kinetics

Details of APO(a) kinetic calculations and the use of stable isotopes have been published [38–40]. Briefly, subjects were given a bolus injection of 5,5,5-D_3_-leucine dissolved in 0.15 M NaCl (10 μmol/kg body weight), followed by a constant infusion of D_3_-leucine dissolved in 0.15 M NaCl (10 μmol/kg body weight/hour) for 15 h. During the study, steady state metabolic conditions were maintained by isocaloric, low-fat liquid meals (57% carbohydrate, 18% fat, 25% protein). The latter, to maintain steady state levels of concentrations of the proteins being studied. EDTA blood samples were collected at different time points over 24 h, and isolated plasma was stored in aliquots at −80 °C. The incorporation of the stable isotope D_3_-leucine into the APO(a) protein was measured as described by Zhou et al. [57]. APO(a) FCR (pools/day) was calculated as the D_3_-leucine enrichment in the APO(a) protein, assessed by linear regression analyses, relative to the asymptotic D_3_-leucine enrichment in VLDL APOB100 in each individual during the 15-h infusion period. APO(a) PR (nmol/kg/day) was calculated by multiplying the FCR and the pool size of APO(a), which is the product of the APO(a) concentration and the plasma volume (taken as 45 mL/kg body weight).

### 2.4. Weighted isoform size (wIS) calculation

We calculated the *wIS* using the estimated isoform size and expression levels obtained via gel electrophoresis method described above. For example, if the two isoforms are size 20 and 30, with an expression of 70% and 30%, the *wIS* is (0.7*20)+(0.3*30) = 23 [35].

### 2.5. IL-6, IL-18, IL-18 binding protein, and hsCRP measurements

Plasma IL-6 (D6050, R&D Systems, Minneapolis, MN), IL-18 (DY318–05, R&D Systems, Minneapolis, MN), and IL-18BP (EHIL18BP, Thermofisher, Waltham, Massachusetts) levels were measured by the Reyes-Soffer laboratory using ELISA kits according to manufacturer’s instructions. HsCRP levels were measured on an Integra400plus (Roche) for plasma samples by the Irving Institute for Clinical and Translational Research Biomarkers laboratory.

### 2.6. Statistical analyses

Data were analyzed using R, version 4.2.2. Variables with a skewed distribution including Lp(a), oxPL bound to APO(a) [oxPL-APO(a)], oxPL bound to APOB100 [oxPL-APOB100], APO(a) PR, APO(a) FCR, IL-6, IL-18, IL-18BP, hsCRP, and *wIS* were subjected to natural logarithm (ln) transformation prior to analysis. A constant of 1 was added to hsCRP before ln-transformation because of zero values among two of the participants.

Spearman coefficients were calculated to determine the magnitude of correlation between plasma Lp(a) levels, *wIS*, oxPL-APO(a), oxPL-APOB100, APO(a) PR, APO(a) FCR, IL-6, IL-18, IL-18BP, and hsCRP. Multivariate linear regression analysis was performed to examine the relationships between the predictors Lp(a), oxPL-APO(a), oxPL-APOB100, APO(a) PR, or APO(a) FCR, and the outcome variables IL-6, IL-18, IL-18BP, or hsCRP. We also assessed the relationships between the predictor wIS, and the outcome variables APO(a) PR or APO(a) FCR, as well as the relationships between the predictors IL-6, IL-18, IL-18BP, or hsCRP, and the outcome variables Lp(a), APO(a) PR, or APO(a) FCR. We adjusted our regression models for the covariates age, sex, SRRE, and ln-transformed *wIS* to examine the contribution of these variables to the associations.

Importantly, stable isotope studies in humans are costly. With our current study sample size we do not have the power to analyse the independent effects of each SRRE. Based on numerous studies that we have completed using stable isotope studies in humans, our difference in FCR between groups (Blacks and Whites) is 0.04 with a standard deviation of 0.08 [38–40], hence we would need 64 subjects in each group (128 total), or 43 Whites and 128 Blacks. Of note, we generally obtain this 1:3 distribution in our cohorts [38–40]. For PR, our observed difference between groups (Blacks and Whites) is 0.39 with a standard deviation of 0.36 [38–40], hence we would need 15 subjects in each group (30 total), or 10 Whites and 29 Blacks. Due to these considerations, we adjusted the associations for SRRE in the current data set.

We present the B-coefficient (regression coefficient) and 95% confidence interval (CI) to describe the associations between predictors and outcome variables. Additionally, B was converted to a percentage change in outcome using the formula: % change = (exp(B)-1)*100. To determine differences between predictors or outcome variables per SRRE category, we performed the Kruskal-Wallis test with post-hoc Dunn’s test for continuous data, and a chi-squared test for categorical data (GraphPad Prism 9.5.0). A *p*-value <0.05 was considered statistically significant. To identify statistical outliers, we used the ROUT test employing Q = 0.1% (GraphPad Prism 9.5.0).

## 3. Results

### 3.1. Baseline characteristics

Plasma IL-6, IL-18, IL-18BP, and hsCRP from the 26 participants at baseline are shown in Supplementary Table S1. We identified one statistical outlier in hsCRP values (13.05 mg/L). After removal, 25 participants were included in the analyses. Table 1 lists the baseline characteristics of the 25 subjects included in this study. The mean age was 48 years and 14 (56%) were female. Ten participants identified themselves as Black (40%), nine as Hispanic (36%), and six as White (24%). Among the Black participants, there were 5 males and 5 females; the Hispanic participants included 2 males and 7 females, and among the White participants, there were 4 males and 2 females. The median plasma Lp(a) level was 52.20 nmol/L (interquartile range [IQR] 37.30–116.40 nmol/L). Consistent with previous studies [58], median Lp(a) levels were numerically higher in Black participants (56.70 [49.88–117.10] nmol/L), compared to Hispanic (42.00 [29.70–116.40] nmol/L) or White participants (49.80 [27.12–62.27] nmol/L), although these differences did not reach statistical significance. Plasma hsCRP levels were elevated in Hispanic participants compared to Black (*p* = 0.0098) and White (*p* = 0.0035) participants. It is unclear why hsCRP plasma levels exceeded 2 mg/L in eight out of nine Hispanic participants; however, similar levels have been found in the large Multi-Ethnic Study of Atherosclerosis (MESA) study [59]. IL-6 and IL-18 plasma levels were in a similar range for all different SRRE. The subjects had median APO(a) PR of 0.51 [0.22–0.65] nmol/kg/day, and APO(a) FCR of 0.17 [0.13–0.22] pools/day. Most subjects expressed two APO(a) isoforms, with predominance of smaller isoforms. Eight individuals (32%) expressed only one APO(a) isoform (Supplementary Table S2). The median *wIS* was 22.63 [19.00–26.32]. Supplementary Table S2 also lists the Lp(a) plasma level, APO(a) PR, and APO(a) FCR for each individual subject.

In line with previous studies [60,61], *wIS* and plasma Lp(a) levels showed a negative correlation (*r* = −0.465, *p* = 0.01947) (Supplementary Table S3). Plasma Lp(a) levels correlated positively with APO(a) PR (*r* = 0.735, *p* < 0.001), and trended towards a negative correlation with APO(a) FCR (*r* = −0.355, *p* = 0.082) (Supplementary Table S3). We found no correlations between *wIS* and APO(a) PR, assessed by Spearman test, in the univariate regression model, or after adjustment for age, sex, and SRRE in multivariate regression analyses. Additionally, in line with previous studies [35], *wIS* and APO(a) FCR showed a modest positive correlation in univariate regression analyses, which was strengthened after adjustment for age and sex, but was not affected by SRRE (Supplementary Table S3 and S4).

### 3.2. Plasma Lp(a) levels, oxPL-APO(a), or oxPL-APOB100 and plasma IL-6, IL-18, IL-18BP, or hsCRP

Plasma Lp(a) levels showed a positive association with oxPL-APO(a) and oxPL-APOB100 (*r* = 0.742, *r* = 0.793; both *p* < 0.001, Supplementary Table S3), similar to findings in larger cohorts [13]. We did not find associations of plasma Lp(a) levels, oxPL-APO(a), or oxPL-APOB100 with plasma IL-6, IL-18BP, or hsCRP, also not after adjustments for age, sex, SRRE or *wIS*. Plasma Lp(a) tended to associate positively with IL-18 (*p* = 0.051, Table 2). Plasma oxPL-APOB100, but not oxPL-APO(a), levels associated positively with plasma IL-18, only after adjusting for *wIS* (Fig. S1, Table 2).

### 3.3. APO(a) kinetics and plasma IL-6

**Fig. 1.**
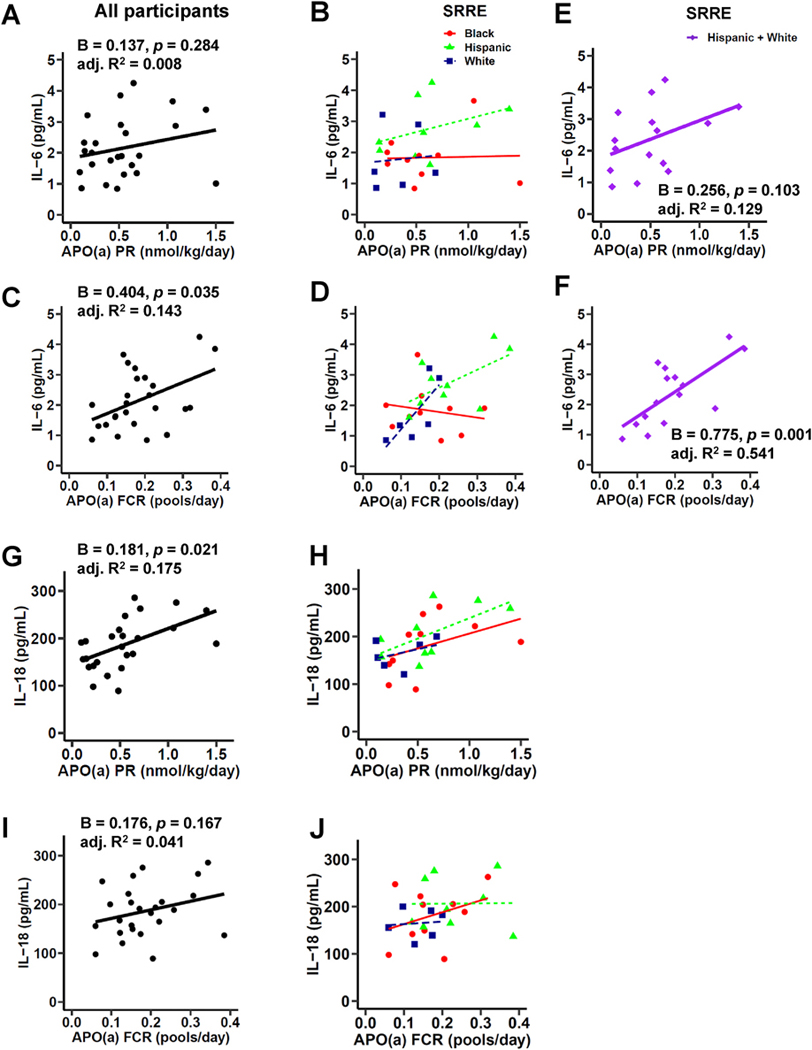
Relationships of APO(a) production rate (PR), or APO(a) fractional catabolic rate (FCR) with plasma IL-6 and IL-18 levels. Results are presented as univariate linear regression plots. (A–F) Relationships of APO(a) PR (A, B, E) or APO(a) FCR (C, D, F) with plasma IL-6 in (A, C) all participants (n = 25), (B, D) separated by three SRRE categories, or (E, F) in Hispanic and White participants combined. (G–J) Relationships of APO(a) PR (G–H) or APO (a) FCR (I–J) with plasma IL-18 levels in (G, I) all participants (n = 25), or (H, J) separated by three SRRE categories. (A, C, E, F, G, I) B-coefficients (regression coefficients), p-values, and adjusted R^2^ values are shown. (B, D, H, J) Red dots, Black participants (n = 10); green triangles, Hispanic participants (n = 9); darkblue squares, White participants (n = 6). (E, F) Purple diamonds, Hispanic and White participants (n = 15). PR, production rate; FCR, fractional catabolic rate; IL-6, interleukin-6; IL-18, interleukin-18. (For interpretation of the references to colour in this figure legend, the reader is referred to the Web version of this article.)

We next focused on examining the associations between APO(a) kinetics (PR and FCR) and plasma IL-6. We found a positive association between APO(a) PR and plasma IL-6 after adjustment for age and sex (B = 0.278, *p* = 0.034), which was independent of *wIS* (B = 0.295, *p* = 0.020). Each one-SD increase in APO(a) PR (0.38 nmol/kg/day) was associated with 34.3% increase in plasma IL-6 levels (B = 0.295, *p* = 0.020) in the model with concomitant adjustments for age, sex, and *wIS* (Table 3). This relationship was dependent on SRRE (B = 0.244, *p* = 0.053) (Fig. 1A, Table 3), which may reflect a stronger association between APO(a) PR and plasma IL-6 in White or Hispanic than in Black participants, even though these associations did not reach statistical significance in the separate SRRE groups (Fig. 1B, Supplementary Table S5), presumably because of the small sample size.

We then examined associations between APO(a) FCR and plasma IL-6. APO(a) FCR showed a positive association with plasma IL-6 after adjustment for age and sex (B = 0.534, *p* = 0.009; Fig. 1C, Table 3), which lost statistical significance after adjustment for SRRE on top of *wIS* (B = 0.391, *p* = 0.086; Table 3), again indicating a major role for SRRE in these relationships. Each one-SD increase in APO(a) FCR (0.086 pools/day) was associated with 68.0% increase in plasma IL-6 levels (B = 0.519, *p* = 0.032) in the model with adjustments for age, sex, and *wIS* (Table 3). Again, associations did not reach statistical significance in separate SRRE groups (Fig. 1D, Supplementary Table S6).

However, since the relationships were dependent on SRRE, and White and Hispanic, but not Black participants, showed a similar trend between APO(a) PR as well as APO(a) FCR and plasma IL-6 levels (Fig. 1B and D, Supplementary Tables S5–S6), we combined White and Hispanic participants for further analysis. Of note, Hispanic participants had higher hsCRP levels than White participants (Table 1). We acknowledge this caveat when combining participants of these two different SRRE. For all other parameters we assessed, we observed no differences between Hispanic and White participants (Table 1). In Hispanic and White participants combined, we found a positive association between APO(a) PR and plasma IL-6 levels after adjustment for age and sex (B = 0.393, *p* = 0.010), which was independent of SRRE and *wIS* (B = 0.410, *p* = 0.019) (Fig. 1E, Table 4). Each one-SD increase in APO(a) PR (0.37 nmol/kg/day) was associated with 50.7% increase in plasma IL-6 levels (B = 0.410, *p* = 0.019) in the model adjusted for age, sex, SRRE, and *wIS* (Table 4). Since APO(a) PR shows a strong correlation with plasma Lp(a) levels (Supplementary Table S3), we then assessed the relationship between plasma Lp(a) and plasma IL-6 in Hispanic and White participants combined. We found a positive association between Lp(a) and plasma IL-6 levels after adjustment for age, sex, and *wIS* (B = 0.436, p = 0.036) (Table 4). In Hispanic and White participants combined, each one-SD increase in Lp(a) (57.4 nmol/L) was associated with 54.7% increase in plasma IL-6 levels in the model adjusted for age, sex, and *wIS*.

Further, in Hispanic and White participants combined, we found a positive association between APO(a) FCR and plasma IL-6 (B = 0.775, *p* = 0.001; Fig. 1F, Table 4), which was independent of age, sex, SRRE and *wIS* (B = 0.956, *p* = 0.010). Each one-SD increase in APO(a) FCR (0.090 pools/day) was associated with 1.6-fold increase in plasma IL-6 levels (B = 0.956, *p* = 0.010) in the model with concomitant adjustments for age, sex, SRRE, and *wIS* (Table 4).

Together, these data indicate that APO(a) PR and APO(a) FCR positively associate with plasma IL-6 in Hispanic and White participants combined, independent of *wIS*. In Black participants, APO(a) PR and APO(a) FCR do not associate with IL-6.

In highly pro-inflammatory conditions such as RA and CKD, IL-6 increases plasma levels of Lp(a) [47,48]. Lp(a) levels are elevated only when plasma IL-6 levels exceed 6 pg/mL [52]. Even though this is more than two-fold higher than the plasma IL-6 levels in the subjects we studied, we cannot completely exclude reverse associations. When examining the relationship between plasma IL-6 and APO(a) kinetics, we found indeed that plasma IL-6 also shows a positive association with APO(a) PR and APO(a) FCR (Supplementary Table S7). Each one-SD increase in plasma IL-6 (0.97 pg/mL) was associated with 1.3-fold increase in APO(a) PR (B = 0.824, *p* = 0.020) and 49.6% increase in APO (a) FCR (B = 0.403, *p* = 0.032), in the model with concomitant adjustments for age, sex, and wIS (Supplementary Table S7). Again, these associations did not reach statistical significance in separate SRRE groups (Supplementary Table S8). In Hispanic and White participants combined, we did find positive associations between plasma IL-6 and APO(a) PR (B = 1.155, p = 0.019) or plasma IL-6 and APO(a) FCR (B = 0.568, p = 0.010; Supplementary Table S9), which were both independent of age, sex, and wIS (Supplementary Table S9). Each one-SD increase in plasma IL-6 (1.05 pg/mL) was associated with 2.3-fold increase in APO(a) PR or 76.5% increase in APO(a) FCR (Supplementary Table S9).

### 3.4. APO(a) kinetics and plasma IL-18, IL-18BP, and hsCRP

We then examined potential associations between APO(a) kinetics, plasma IL-18, IL-18BP, and hsCRP in the whole cohort of 25 participants including White, Hispanic, and Black participants. We found a positive association between APO(a) PR and plasma IL-18 (B = 0.181, *p* = 0.021; Fig. 1G, Table 3), independent of age, sex, SRRE, and *wIS* (B = 0.259, *p* = 0.013). Each one-SD increase in APO(a) PR (0.38 nmol/kg/day) was associated with 29.6% increase in plasma IL-18 levels (B = 0.259, *p* = 0.013) in the model with concomitant adjustments for age, sex, SRRE, and *wIS* (Table 3). In all SRRE categories, we observed a tendency towards a positive relationship between APO(a) PR and plasma IL-18 levels (Fig. 1H–Supplementary Table S5), which did not reach statistical significance for the SRRE categories separately. APO(a) PR did not show any associations with plasma IL-18BP or hsCRP levels (Figs. S2A and S2C, Table 3) in the whole cohort, and also not in separate SRRE categories (Figs. S2B and S2D, Supplementary Table S5).

Further, APO(a) FCR did not show any associations with plasma IL-18, IL-18BP or hsCRP in the whole cohort or among different SRRE categories (Fig. 1I–J, Supplementary Fig. S2E–H, Table 3, Supplementary Table S6). Together, these data indicate that in terms of APO(a) kinetics, APO(a) PR associates positively with plasma IL-18 independent of *wIS* and SRRE.

For IL-18, IL-18BP, and hsCRP, we also examined reverse associations, *i.e.* whether these cytokines were positively associated with APO (a) PR and APO(a) FCR. We found that plasma IL-18 levels positively associated with APO(a) PR, but not with APO(a) FCR, in the model adjusted for age, sex, SRRE, and *wIS* (B = 1.139, p = 0.013; Supplementary Table S7). Each one-SD increase in plasma IL-18 (53.3 pg/mL) was associated with 2.1-fold increase in APO(a) PR (Supplementary Table S7). Again, we did not find any associations between IL-18BP or hsCRP and APO(a) PR or APO(a) FCR in the whole cohort (Supplementary Table S7) or in separate SRRE groups (Supplementary Table S8).

## 4. Discussion

Previous studies have shown that Lp(a) enhances the expression of pro-inflammatory interleukins in monocytes and macrophages [16, 21–27], as well as vascular inflammation. OxPL bound to Lp(a) have been proposed as a driver of inflammation [16,23,27]. Substantiating these data, we now show an APO(a) isoform size dependent-positive association between plasma oxPL-APOB100 and plasma IL-18. Previous studies have shown that up to 85% of the oxPL-APOB100 in plasma can be part of the circulating Lp(a) particle [15]. Based on this, it may be that most of the oxPL in our cohort were on the Lp(a) particles. Therefore, our data may suggest an APO(a) isoform size dependent-positive association between oxPL-APOB100 on Lp(a) and plasma IL-18.

**Fig. 2.**
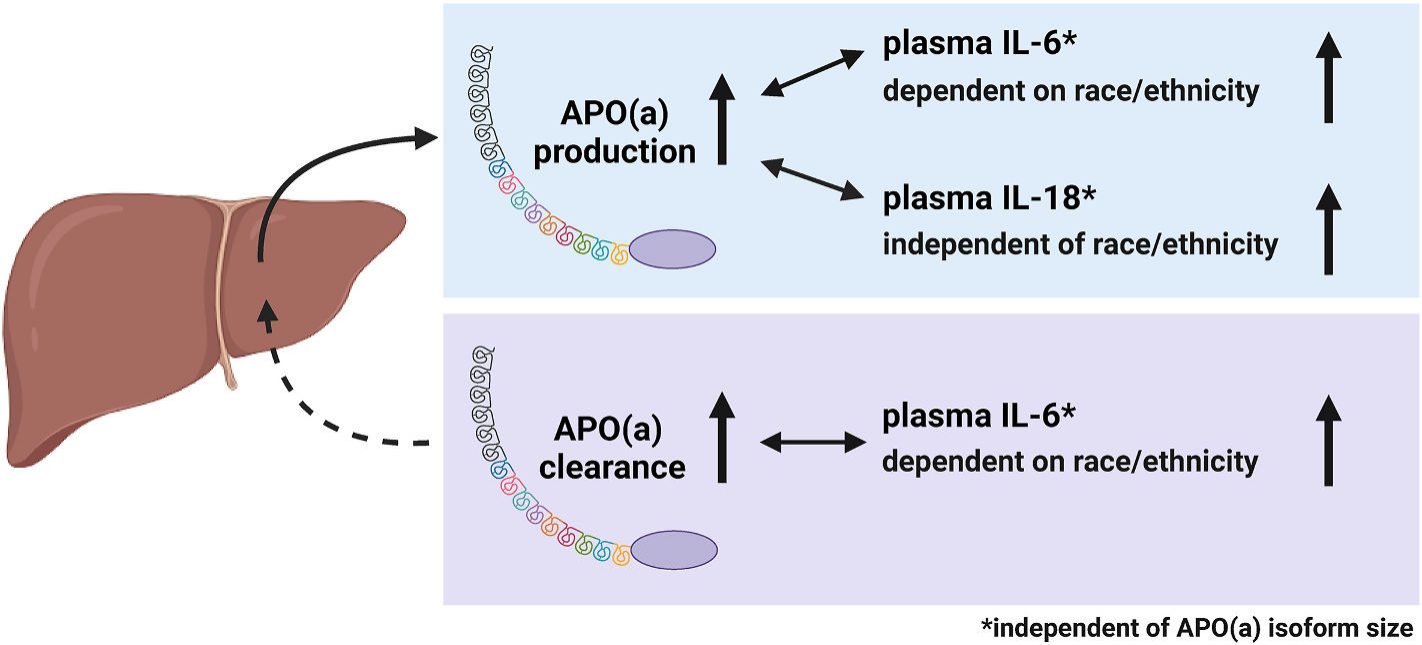
Graphical abstract. APO(a) production and clearance are positively associated with plasma IL-6, dependent on race/ethnicity and independent of APO(a) isoform size. APO(a) production is positively associated with plasma IL-18, independent of race/ethnicity and APO(a) isoform size. As indicated by the arrows, the associations are in both directions.

Importantly, we found positive associations between APO(a) PR and plasma levels of IL-18 or IL-6. While both relationships were independent of APO(a) isoform size, the relationship between APO(a) PR and plasma IL-6 was dependent on SRRE, and only observed in Hispanic and White, but not Black participants. In addition, APO(a) FCR associated positively with plasma IL-6 in Hispanic and White, but not Black participants, independent of APO(a) isoform size. Our findings, summarized in the graphical abstract (Fig. 2) suggest a positive relationship between APO(a) kinetics and plasma IL-6 levels in Hispanic and White participants and substantiate the important role of including racial diversity when studying APO(a) kinetics [35]. It may seem counterintuitive that both APO(a) PR and APO(a) FCR show a positive association with plasma IL-6 levels in Hispanic and White participants combined. However, it should be noted, that both the PR and the FCR regulate plasma levels of Lp(a) [35]. Hence, we conclude that IL-6 can be linked to both mechanisms.

The main clinical application of our findings relates to the ongoing novel therapeutics targeting APO(a) PR [6–11]. Our data suggest that these drugs may decrease ASCVD by reducing Lp(a) and pro-inflammatory interleukins such as IL-6 and IL-18.

IL-18 has long been known as interferon (IFN)γ inducing factor [45], and therefore, it is of interest that antisense oligonucleotides targeting APO(a) lowered plasma Lp(a) levels by ~47% and decreased IFNγ responsive mRNA expression in blood monocytes [29]. These data suggest that in the setting of enhanced APO(a) PR, IL-18 may increase IFNγ producing T-cells, and consequently IFNγ responsive mRNA expression in blood monocytes. Of note, IL-12 also critically increases IFNγ expression in T-cells [45]; however, we did not measure IL-12 plasma levels in our cohort. The mechanisms that underlie the association of APO(a) PR with increased plasma IL-18 will be topic of future study.

Participants in our study did not show a hyper-inflammatory state that is associated with elevated plasma Lp(a) as observed in COVID-19, RA, or CKD, where the increase in plasma Lp(a) is IL-6 dependent [47, 48,51]. Previous studies have shown that plasma Lp(a) levels were elevated only when plasma IL-6 levels were above 6 pg/mL [52], which is more than two-fold higher than the plasma IL-6 levels in our cohort. Therefore, our *a priori* hypothesis focused on APO(a) kinetics as predictors and plasma IL-6 or IL-18 levels as outcome variables in our study. Even so, we did examine reverse associations, *i.e.* association between IL-6, IL-18, and APO(a) PR, and APO(a) FCR. Plasma IL-6 was positively associated with APO(a) PR and APO(a) FCR in Hispanic and White, but not Black participants. This may be consistent with IL-6 response elements in the APO(a) promoter, as proposed previously in a study carried out in White participants [52,53]. *LPA* single nucleotide polymorphisms differ among ethnic groups [37,62], which may account for the racial differences in terms of associations.

Plasma IL-18 was positively associated with APO(a) PR in all participants independent of SRRE. This may simply reflect an association between inflammatory cytokines and Lp(a). Indeed, elevated plasma tumor necrosis factor (TNF)α has also been positively associated with plasma Lp(a) levels, but TNFα blockade by adalimumab did not decrease plasma Lp(a) levels in RA patients, while the IL-6 receptor inhibitor tocilizumab lowered plasma Lp(a) in RA [47].

Despite our data showing new links between APO(a) kinetics and plasma IL-18 and IL-6 in an ethnically diverse cohort, our study has some limitations. We acknowledge the potential conflict of structural and social factors in self-reported race. Other limitations include the measurement of only a select group of inflammatory markers (IL-6, IL-18, IL-18BP, and hsCRP), that although carefully chosen, limited unbiased exploration of other pro-inflammatory cytokines. Furthermore, the number of individuals in our dataset was limited, being the consequence of the sample size that can be feasibly achieved when conducting kinetic studies.

## Supplementary Material

Figure S1-S2 and all supplementary tables

## Figures and Tables

**Table 1:** Characteristics of 25 participants included in the analyses.

Characteristics	All participants (n = 25)	Black participants (n = 10)	Hispanic participants (n = 9)	White participants (n = 6)	*p*-value		
					
					Black *vs* Hispanic	Black *vs* White	Hispanic *vs* White

Age (years), mean (SD)	48 (12)	46 (13)	43 (11)	57 (11)	>0.999	0.310	0.121
Sex (female), n Male	11	5	2	4	0.210	0.515	0.085
Female	14	5	7	2			
BMI (kg/m^2^), median (IQR)	28.8 (25.7, 31.3)	29.6 (26.5, 32.6)	29.5 (28.2, 32.8)	23.6 (22.6, 25.8)	>0.999	0.070	0.063
Glucose (mg/dL), median (IQR)	88.0 (83.0, 92.0)	85.5 (80.3, 91.5)	88.0 (85.0, 93.0)	88.5 (87.3, 91.3)	>0.999	0.897	>0.999
Lp(a) (nmol/L), median (IQR)	52.20 (37.30, 116.40)	56.70 (49.88, 117.10)	42.00 (29.70, 116.40)	49.80 (27.12, 62.27)	0.888	0.927	>0.999
Oxidized phospholipids APO(a) (relative light units (RLU)), median (IQR)	18.05 (5.63, 32.39)	18.67 (7.58, 32.32)	17.15 (6.76, 33.69)	12.96 (5.21, 24.44)	>0.999	>0.999	>0.999
Oxidized phospholipids APOB100 (relative light units (RLU)), median (IQR)	4.53 (3.63, 6.28)	5.04 (4.20, 6.38)	4.39 (3.63, 6.08)	3.89 (3.32, 5.78)	>0.999	0.807	>0.999
APO(a) production rate (nmol/kg/day), median (IQR)	0.51 (0.22, 0.65)	0.50 (0.30, 0.67)	0.57 (0.49, 0.65)	0.27 (0.13, 0.48)	>0.999	0.422	0.317
APO(a) fractional catabolic rate (pools/day), median (IQR)	0.17 (0.13, 0.22)	0.15 (0.13, 0.22)	0.21 (0.16, 0.31)	0.15 (0.10, 0.17)	0.503	>0.999	0.160
Weighted iso form size, median (IQR)	22.63 (19.00, 26.32)	23.55 (20.78, 26.83)	21.12(19.00, 26.20)	22.59 (18.80, 25.80)	>0.999	0.971	>0.999
Interleukin-6 (pg/mL), median (IQR)	1.91 (1.38, 2.87)	1.83 (1.38, 1.98)	2.63 (2.06, 3.39)	1.36 (1.05, 2.52)	0.118	>0.999	0.130
Interleukin-18 (pg/mL), median (IQR)	188.72 (149.845, 218.00)	196.35 (143.77, 217.68)	193.80 (164.60, 258.90)	169.10 (143.50 189.00)	>0.999	>0.999	0.409
Interleukin-18 binding protein (ng/mL), median (IQR)	48.36 (32.46, 79.00)	64.63 (47.74, 90.62)	48.36 (35.43, 71.79)	28.65 (25.61, 42.55)	>0.999	0.095	0.521
High sensitivity C reactive protein (mg/L), median (IQR)	1.14 (0.75, 2.77)	0.83 (0.47, 1.58)	2.89 (2.59, 7.27)	0.82 (0.43, 0.96)	**0.0098** ***	>0.999	**0.0035** **

SD, standard deviation; IQR, interquartile range. Asterisks (**) indicate *p*-values

** *p* < 0.01

*** *p* < 0.001.

**Table 2:** Relationships of plasma Lp(a) levels, oxidized phospholipids (oxPL) on APO(a) [oxPL-APO(a)], or oxPL on APOB100 [oxPL-APOB100] with plasma IL-6, IL-18, IL-18BP, or hsCRP.

Outcome		Predictor: Lp(a)		Predictor: oxPL-APO(a)		Predictor: oxPL-APOB100	
			
	Model	B (95% CI)	*p*-value	B (95% CI)	*p*-value	B (95% CI)	*p*-value

**IL-6**	univariate	−0.030 (−0.038, −0.022)	0.817	−0.067 (−0.073, −0.061)	0.481	0.001 (−0.012, 0.015)	0.995
	+ age, sex	0.075 (0.067, 0.084)	0.594	−0.016 (−0.022, −0.010)	0.873	0.090 (0.076, 0.103)	0.679
	+ age, sex, SRRE	−0.008 (−0.017, 0.000)	0.950	−0.038 (−0.043, −0.032)	0.657	−0.019 (−0.031, −0.006)	0.923
	+ age, sex, *wIS*	0.191 (0.182, 0.201)	0.201	0.065 (0.058, 0.071)	0.553	0.335 (0.320, 0.350)	0.166
	+ age, sex, SRRE, *wIS*	0.190 (0.180, 0.200)	0.245	0.041 (0.035, 0.047)	0.671	0.289 (0.274, 0.304)	0.233
**IL-18**	univariate	0.110 (0.105, 0.115)	0.182	0.077 (0.074, 0.081)	0.202	0.224 (0.216, 0.232)	0.092
	+ age, sex	0.172 (0.166, 0.178)	0.066	0.098 (0.094, 0.102)	0.135	0.278 (0.269, 0.286)	0.051
	+ age, sex, SRRE	0.174 (0.167, 0.180)	0.095	0.092 (0.088, 0.096)	0.168	0.261 (0.252, 0.270)	0.082
	+ age, sex, *wIS*	0.204 (0.197, 0.210)	0.053	0.124 (0.119, 0.128)	0.106	0.370 (0.360, 0.379)	**0.028** *
	+ age, sex, SRRE, *wIS*	0.265 (0.257, 0.272)	0.051	0.119 (0.114, 0.124)	0.141	0.419 (0.407, 0.430)	**0.036** *
**IL-18BP**	univariate	0.139 (0.128, 0.149)	0.316	0.178 (0.170, 0.186)	0.161	0.388 (0.370, 0.405)	0.169
	+ age, sex	0.215 (0.203, 0.228)	0.291	0.211 (0.203, 0.219)	0.128	0.466 (0.447, 0.484)	0.129
	+ age, sex, SRRE	0.095 (0.081, 0.109)	0.665	0.193 (0.185, 0.202)	0.154	0.369 (0.350, 0.388)	0.238
	+ age, sex, *wIS*	0.266 (0.252, 0.280)	0.246	0.280 (0.270, 0.289)	0.082	0.649 (0.627, 0.671)	0.075
	+ age, sex, SRRE, *wIS*	0.030 (0.012, 0.048)	0.918	0.214 (0.204, 0.225)	0.194	0.442 (0.416, 0.468)	0.295
**hsCRP**	univariate	0.176 (0.165, 0.186)	0.349	0.149 (0.141, 0.157)	0.246	0.322 (0.305, 0.340)	0.258
	+ age, sex	0.349 (0.337, 0.360)	0.068	0.190 (0.182, 0.199)	0.155	0.489 (0.472, 0.507)	0.095
	+ age, sex, SRRE	0.276 (0.268, 0.285)	0.054	0.157 (0.152, 0.163)	0.086	0.348 (0.336, 0.361)	0.097
	+ age, sex, *wIS*	0.333 (0.320, 0.346)	0.119	0.171 (0.161, 0.181)	0.271	0.477 (0.456, 0.498)	0.171
	+ age, sex, SRRE, *wIS*	0.216 (0.205, 0.228)	0.236	0.110 (0.103, 0.117)	0.305	0.226 (0.209, 0.243)	0.410

Asterisks (*) indicate *p*-values

* *p* < 0.05. oxPL, oxidized phospholipids; IL-6, interleukin-6; IL-18, interleukin-18; IL-18BP, IL-18 binding protein; hsCRP, high sensitivity C-reactive protein; SRRE, self-reported race and ethnicity; *wIS*, weighted isoform size.

**Table 3:** Relationships of APO(a) production rate and APO(a) fractional catabolic rate with plasma IL-6, IL-18, IL-18BP, or hsCRP.

Outcome		Predictor: APO(a) PR			Predictor: APO(a) FCR		
			
	Model	B (95% CI)	Adj. R^2^	*p*-value	B (95% CI)	Adj. R^2^	*p*-value

**IL-6**	univariate	0.137 (0.129, 0.145)	0.008	0.284	0.404 (0.392, 0.415)	0.143	**0.035** *
	+ age, sex	0.278 (0.270, 0.286)	0.209	**0.034** *	0.534 (0.522, 0.546)	0.292	**0.009** **
	+ age, sex, SRRE	0.186 (0.178, 0.194)	0.350	0.153	0.424 (0.413, 0.434)	0.455	**0.021** *
	+ age, sex, *wIS*	0.295 (0.288, 0.302)	0.289	**0.020** *	0.519 (0.505, 0.534)	0.257	**0.032** *
	+ age, sex, SRRE, *wIS*	0.244 (0.236, 0.251)	0.452	0.053	0.391 (0.377, 0.405)	0.427	0.086
**IL-18**	univariate	0.181 (0.177, 0.186)	0.175	**0.021** *	0.176 (0.168, 0.184)	0.041	0.167
	+ age, sex	0.242 (0.238, 0.247)	0.236	**0.005** **	0.204 (0.195, 0.213)	−0.021	0.174
	+ age, sex, SRRE	0.251 (0.245, 0.256)	0.197	**0.012** *	0.167 (0.157, 0.177)	−0.065	0.288
	+ age, sex, *wIS*	0.243 (0.238, 0.248)	0.198	**0.006** **	0.298 (0.287, 0.309)	−0.018	0.097
	+ age, sex, SRRE, *wIS*	0.259 (0.253, 0.265)	0.160	**0.013** *	0.289 (0.277, 0.301)	−0.055	0.144
**IL-18BP**	univariate	0.083 (0.072, 0.094)	−0.033	0.635	−0.133 (−0.150, −0.116)	−0.032	0.625
	+ age, sex	0.125 (0.112, 0.137)	−0.113	0.534	−0.209 (−0.229, −0.189)	−0.111	0.517
	+ age, sex, SRRE	0.009 (−0.005, 0.023)	−0.065	0.966	−0.189 (−0.209, −0.169)	−0.046	0.558
	+ age, sex, *wIS*	0.126 (0.113, 0.139)	−0.168	0.540	−0.303 (−0.327, −0.279)	−0.155	0.437
	+ age, sex, SRRE, *wIS*	−0.020 (−0.034, −0.005)	−0.105	0.931	−0.127 (−0.153, −0.101)	−0.100	0.758
**hsCRP**	univariate	0.207 (0.197, 0.218)	0.021	0.231	0.082 (0.065, 0.099)	−0.039	0.765
	+ age, sex	0.296 (0.285, 0.308)	0.080	0.115	−0.091 (−0.110, −0.071)	−0.035	0.771
	+ age, sex, SRRE	0.104 (0.094, 0.113)	0.518	0.488	−0.376 (−0.389, −0.363)	0.582	0.078
	+ age, sex, *wIS*	0.284 (0.273, 0.296)	0.066	0.134	0.082 (0.059, 0.105)	−0.046	0.825
	+ age, sex, SRRE, *wIS*	0.053 (0.044, 0.063)	0.548	0.719	−0.268 (−0.284, −0.251)	0.571	0.307

Asterisks (*) indicate *p*-values

* *p* < 0.05

** *p* < 0.01.

PR, production rate; FCR, fractional catabolic rate.

**Table 4:** Relationships of APO(a) production rate, APO(a) fractional catabolic rate, and plasma Lp(a) levels with plasma IL-6 in Hispanic and White participants combined.

		Hispanic + White			Hispanic + White			Hispanic + White		
			
Outcome		Predictor: APO(a) PR			Predictor: APO(a) FCR			Predictor: Lp(a)		
			
	Model	B (95% CI)	Adj. R^2^	*p*-value	B (95% CI)	Adj. R^2^	*p*-value	B (95% CI)	Adj. R^2^	*p*-value

**IL-6**	univariate	0.256 (0.246, 0.265)	0.129	0.103	0.775 (0.763, 0.786)	0.541	**0.001** ***	0.002 (−0.008, 0.013)	−0.077	0.988
	+ age, sex	0.393 (0.385, 0.401)	0.483	**0.010** **	0.983 (0.964, 1.001)	0.530	**0.006** **	0.299 (0.287, 0.313)	0.220	0.126
	+ age, sex, SRRE	0.349 (0.339, 0.358)	0.451	**0.043** *	0.915 (0.898, 0.931)	0.626	**0.005** **	0.191 (0.177, 0.204)	0.218	0.389
	+ age, sex, *wIS*	0.434 (0.426, 0.441)	0.572	**0.004** **	1.021 (1.000, 1.042)	0.487	**0.011** *	0.436 (0.425, 0.448)	0.358	**0.036** *
	+ age, sex, SRRE, *wIS*	0.410 (0.401, 0.420)	0.529	**0.019** *	0.956 (0.937, 0.975)	0.590	**0.010** **	0.375 (0.360, 0.390)	0.301	0.146

n = 15. Asterisks (*) indicate *p*-values

* *p* < 0.05

** *p* < 0.01

*** *p* < 0.001.

## Data Availability

Data and code to reproduce the analyses of this study are available from the corresponding authors upon reasonable request.

## Appendix A. Supplementary data

Supplementary data to this article can be found online at https://doi.org/10.1016/j.atherosclerosis.2024.117474.
