# Supplementary material for: Apolipoprotein(a) production and clearance are associated with plasma IL-6 and IL-18 levels, dependent on ethnicity
Source: Atherosclerosis. Author manuscript; Available in PMC 2026 Mar 30. (PMC13034173; doi:10.1016/j.atherosclerosis.2024.117474)
Supplement: Figure S1-S2 and all supplementary tables [file NIHMS2099605-supplement-Figure_S1-S2_and_all_supplementary_tables.pdf]

SUPPLEMENTAL MATERIAL

Apolipoprotein(a) production and clearance are associated with plasma IL-6 and IL-18 levels, dependent on ethnicity

Anouk G. Groenen[1,*], Anastasiya Matveyenko[2,*], Nelsa Matienzo[2], Benedek Halmos[1], Hanrui Zhang[3], Marit Westerterp[1,†], and Gissette Reyes-Soffer[2,†]

[1]Department of Pediatrics, University Medical Center Groningen, University of Groningen, Groningen, The Netherlands

Columbia University Irving Medical Center, College of Physicians and Surgeons, Department of Medicine, [2]Division of Preventive Medicine and Nutrition, and [3]Division of Cardiology, New York, NY, USA

[*,†] These authors contributed equally to this work

Table S1. Baseline information of study population.

Participant	Age	Sex	SRRE	Interleukin-6 (pg/mL)	Interleukin-18 (pg/mL)	Interleukin-18 binding protein (ng/mL)	High sensitivity C reactive protein (mg/L)
**1**	68	Male	White	0.89	155.77	27.22	1.14
**2**	54	Male	White	1.34	200.16	25.07	0.98
**3**	26	Male	Black	0.84	89.11	48.72	0.00**
**4**	61	Female	Black	1.63	141.88	79.00	0.76
**5**	62	Female	White	3.21	139.36	108.22	0.00**
**6**	56	Male	White	2.90	182.37	46.70	0.75
**7**	49	Female	Black	1.30	247.40	52.27	1.04
**8**	56	Female	Hispanic	2.63	164.63	71.79	2.54
**9**	29	Male	Hispanic	1.60	167.16	35.43	7.27
**10**	50	Female	Black	1.91	262.79	76.99	0.46
**11**	31	Female	Hispanic	1.87	218.00	32.46	1.66
**12**	42	Male	Black	2.31	149.45	110.11	0.41
**13**	29	Female	Hispanic	2.33	193.80	46.94	2.59
**14**	32	Male	Black	1.01	188.72	94.49	0.51
**15**	60	Female	Hispanic	2.06	157.04	62.45	2.89
**16**	39	Female	Black	1.76	203.98	34.84	3.94
**17**	38	Male	Black	1.90	205.25	47.42	0.90
**18**	56	Female	Black	2.00	97.87	139.89	2.62
19	*60*	*Female*	*Black*	*3.88*	*324.68*	*113.43*	***13.05****
**20**	62	Female	White	1.38	191.26	17.52	0.88
**21**	37	Male	White	0.96	120.47	30.08	0.32
**22**	44	Female	Hispanic	2.87	275.64	108.92	7.70
**23**	49	Male	Hispanic	3.39	258.94	97.09	5.12
**24**	46	Female	Hispanic	4.25	285.93	10.16	2.77
**25**	67	Male	Black	3.66	221.82	29.48	1.76
**26**	46	Female	Hispanic	3.85	136.84	48.36	7.31

*Statistical outlier in hsCRP values based on ROUT test employing Q=0.1%. Participant was excluded from the analyses.
**Not detected. These values were reported as 0 for statistical analyses. For further details on statistical analyses, please see the Materials and Methods section.

Table S2. Plasma Lp(a) levels, APO(a) PR, APO(a) FCR, APO(a) isoforms, and *wIS* of subjects included in the analyses.

Participant	Lp(a) (nmol/L)	APO(a) PR (nmol/kg/day)	APO(a) FCR (pools/day)	*wIS*	Smaller APO(a) isoform	% smaller	Larger APO(a) isoform	% larger
**1**	41.9	0.11	0.06	26.7	26	65	28	35
**2**	155.8	0.68	0.10	15.0	15	100		
**3**	52.2	0.48	0.21	19.0	19	100		
**4**	40.5	0.22	0.12	26.3	24	42	28	58
**5**	22.2	0.17	0.17	26.0	26	100		
**6**	57.7	0.52	0.20	25.2	23	80	34	20
**7**	158.3	0.55	0.08	18.8	18	61	20	39
**8**	57.1	0.57	0.22	20.9	20	70	23	30
**9**	116.4	0.63	0.12	17.0	17	100		
**10**	49.4	0.71	0.32	28.1	27	44	29	56
**11**	35.4	0.49	0.31	24.3	19	12	25	88
**12**	37.3	0.26	0.15	24.5	22	18	25	82
**13**	14.7	0.14	0.21	21.1	20	84	27	16
**14**	129.2	1.50	0.26	21.5	20	50	23	50
**15**	21.5	0.15	0.15	26.2	21	60	34	40
**16**	61.2	0.41	0.15	20.5	20	73	22	27
**17**	51.3	0.53	0.23	27.0	27	100		
**18**	41.9	0.22	0.06	22.6	22	79	25	21
**20**	80.8	0.10	0.17	20.0	19	67	22	33
**21**	12.4	0.37	0.13	18.4	18	90	22	10
**22**	63.8	1.08	0.18	19.0	19	100		
**23**	134.4	1.40	0.16	18.0	18	100		
**24**	199.7	0.65	0.34	28.0	28	100		
**25**	42.0	1.05	0.14	27.4	26	55	29	45
**26**	164.4	0.51	0.39	27.4	23	60	34	40

wIS, weighted isoform size.

Table S3. Spearman correlations (*r*) between each pair of continuous predictor variables.

	Age	*wIS*	Lp(a)	oxPL-APO(a)	oxPL-APOB100	APO(a) PR	APO(a) FCR	IL-6	IL-18	IL-18BP	hsCRP
**Age**	1	0.358	-0.095	-0.224	0.044	-0.230	-0.357	0.227	-0.057	-0.018	-0.049
wIS	0.358	1	**-0.465***	**-0.531****	-0.331	-0.123	0.408*	**0.431***	0.030	-0.001	-0.081
**Lp(a)**	-0.095	**-0.465***	1	**0.742*****	**0.793*****	**0.735*****	-0.355	-0.094	0.322	0.085	0.178
**oxPL-APO(a)**	-0.224	**-0.531****	**0.742*****	1	**0.838*****	**0.583****	-0.252	-0.167	0.281	0.240	0.272
**oxPL-APOB100**	0.044	-0.331	**0.793*****	**0.838*****	1	**0.645*****	-0.278	0.060	0.302	0.317	0.327
**APO(a) PR**	-0.230	-0.123	**0.735*****	**0.583****	**0.645*****	1	0.279	0.222	**0.595****	0.092	0.210
**APO(a) FCR**	-0.357	**0.408***	-0.355	-0.252	-0.278	0.279	1	**0.418***	0.255	0.038	-0.023
**IL-6**	0.227	**0.431***	-0.094	-0.167	0.060	0.222	0.418*	1	0.280	0.200	**0.422***
**IL-18**	-0.057	0.030	0.322	0.281	0.302	**0.595****	0.255	0.280	1	-0.215	0.343
**IL-18BP**	-0.018	-0.001	0.085	0.240	0.317	0.092	0.038	0.200	-0.215	1	-0.059
**hsCRP**	-0.049	-0.081	0.178	0.272	0.327	0.210	-0.023	**0.422***	0.343	-0.059	1

Asterisks (*) indicate *p*-values, *p<0.05, **p<0.01, ***p<0.001. *wIS*, weighted isoform size; oxPL, oxidized phospholipids; PR, production rate; FCR, fractional catabolic rate; IL-6, interleukin-6; IL-18, interleukin-18; IL-18BP, IL-18 binding protein; hsCRP, high sensitivity C-reactive protein.

Table S4. Relationships of *wIS* with APO(a) production rate or APO(a) fractional catabolic rate.

**Outcome:**		**Predictor: *wIS***		
	**Model**	**B (95% CI)**	**Adj. R^2**	***p*-value**
**APO(a) PR**	univariate	-0.975 (-1.029, -0.920)	0.012	0.268
	+ age, sex	-0.335 (-0.393, -0.277)	0.062	0.718
	+ age, sex, SRRE	-0.905 (-0.961, -0.849)	0.221	0.316
**APO(a) FCR**	univariate	0.938 (0.904, 0.971)	0.081	0.091
	+ age, sex	1.397 (1.367, 1.428)	0.367	**0.009****
	+ age, sex, SRRE	1.585 (1.554, 1.617)	0.408	**0.004****

Asterisks (*) indicate *p*-values, ***p*<0.01. SRRE, self-reported race and ethnicity.

Table S5. Relationships of APO(a) production rate with plasma IL-6, IL-18, IL-18BP, or hsCRP per SRRE category.

		Black		Hispanic		White	
**Outcome:**		**Predictor: APO(a) PR**		**Predictor: APO(a) PR**		**Predictor: APO(a) PR**	
	**Model**	**B (95% CI)**	***p*-value**	**B (95% CI)**	***p*-value**	**B (95% CI)**	***p*-value**
**IL-6**	univariate	-0.071 (-0.085, -0.056)	0.765	0.165 (0.155, 0.174)	0.301	0.132 (0.110, 0.155)	0.709
	+ age, sex	-0.071 (-0.083, -0.060)	0.697	0.236 (0.225, 0.247)	0.209	0.698 (0.669, 0.726)	0.228
	+ age, sex, *wIS*	-0.067 (-0.078, -0.055)	0.715	0.268 (0.257, 0.279)	0.180	0.874 (0.865, 0.882)	0.077
**IL-18**	univariate	0.321 (0.310, 0.332)	0.098	0.176 (0.168, 0.183)	0.158	0.033 (0.025, 0.041)	0.799
	+ age, sex	0.433 (0.421, 0.445)	0.057	0.219 (0.210, 0.228)	0.187	0.178 (0.165, 0.191)	0.433
	+ age, sex, *wIS*	0.438 (0.426, 0.450)	0.063	0.215 (0.205, 0.226)	0.256	0.122 (0.107, 0.137)	0.645
**IL-18BP**	univariate	-0.315 (-0.332, -0.299)	0.253	0.131 (0.109, 0.153)	0.712	0.082 (0.056, 0.107)	0.842
	+ age, sex	-0.320 (-0.341, -0.298)	0.363	0.019 (-0.009, 0.048)	0.966	0.452 (0.401, 0.502)	0.591
	+ age, sex, *wIS*	-0.316 (-0.339, -0.293)	0.408	-0.113 (-0.135, -0.092)	0.742	0.719 (0.676, 0.763)	0.417
**hsCRP**	univariate	-0.129 (-0.146, -0.112)	0.633	0.283 (0.270, 0.295)	0.179	-0.003 (-0.015, 0.009)	0.986
	+ age, sex	0.026 (0.008, 0.045)	0.929	0.204 (0.188, 0.220)	0.442	-0.066 (-0.084, -0.047)	0.824
	+ age, sex, *wIS*	0.023 (0.003, 0.043)	0.944	0.192 (0.173, 0.210)	0.526	-0.153 (-0.172, -0.133)	0.650

Black, n = 10; Hispanic, n = 9; White, n = 6.

Table S6. Relationships of APO(a) fractional catabolic rate with plasma IL-6, IL-18, IL-18BP, or hsCRP per SRRE category.

		Black		Hispanic		White	
**Outcome:**		**Predictor: APO(a) FCR**		**Predictor: APO(a) FCR**		**Predictor: APO(a) FCR**	
	**Model**	**B (95% CI)**	***p*-value**	**B (95% CI)**	***p*-value**	**B (95% CI)**	***p*-value**
**IL-6**	univariate	-0.147 (-0.165, -0.129)	0.612	0.468 (0.451, 0.485)	0.115	0.930 (0.904, 0.957)	0.079
	+ age, sex	0.094 (0.078, 0.109)	0.705	0.716 (0.696, 0.736)	0.066	1.254 (1.213, 1.295)	0.162
	+ age, sex, *wIS*	-0.243 (-0.267, -0.219)	0.527	0.914 (0.886, 0.942)	0.095	1.206 (1.139, 1.273)	0.393
**IL-18**	univariate	0.219 (0.203, 0.234)	0.390	0.014 (-0.003, 0.030)	0.958	0.025 (0.011, 0.040)	0.913
	+ age, sex	0.445 (0.426, 0.464)	0.179	0.068 (0.042, 0.094)	0.869	0.205 (0.181, 0.229)	0.605
	+ age, sex, *wIS*	0.650 (0.617, 0.682)	0.243	0.265 (0.228, 0.302)	0.657	0.372 (0.364, 0.381)	0.175
**IL-18BP**	univariate	-0.175 (-0.197, -0.153)	0.618	-0.898 (-0.935, -0.860)	0.165	0.542 (0.499, 0.585)	0.450
	+ age, sex	-0.189 (-0.220, -0.159)	0.698	-1.076 (-1.132, -1.021)	0.259	0.622 (0.536, 0.708)	0.660
	+ age, sex, *wIS*	-0.841 (-0.888, -0.795)	0.288	-0.056 (-0.119, 0.008)	0.956	0.270 (0.153, 0.388)	0.886
**hsCRP**	univariate	-0.481 (-0.499, -0.463)	0.120	-0.338 (-0.364, -0.312)	0.433	-0.328 (-0.346, -0.309)	0.299
	+ age, sex	-0.311 (-0.335, -0.287)	0.427	-0.001 (-0.040, 0.038)	0.998	-0.056 (-0.087, -0.026)	0.909
	+ age, sex, *wIS*	-0.389 (-0.431, -0.348)	0.563	0.262 (0.205, 0.318)	0.772	0.092 (0.054, 0.130)	0.880

Black, n = 10; Hispanic, n = 9; White, n = 6.

Table S7. Relationships of plasma IL-6, IL-18, IL-18BP, and hsCRP with plasma Lp(a) levels, APO(a) PR, or APO(a) FCR.

**Outcome:**		**Predictor: IL-6**			**Predictor: IL-18**		
	**Model**	**B (95% CI)**	**Adj. R^2**	***p*-value**	**B (95% CI)**	**Adj. R^2**	***p*-value**
**Lp(a)**	univariate	-0.079 (-0.100, -0.058)	-0.041	0.817	0.692 (0.660, 0.723)	0.036	0.182
	+ age, sex	0.183 (0.161, 0.204)	0.105	0.594	0.880 (0.851, 0.909)	0.230	0.066
	+ age, sex, SRRE	-0.025 (-0.050, 0.000)	0.168	0.950	0.804 (0.775, 0.834)	0.284	0.095
	+ age, sex, *wIS*	0.420 (0.400, 0.440)	0.279	0.201	0.858 (0.831, 0.884)	0.353	0.053
	+ age, sex, SRRE, *wIS*	0.390 (0.370, 0.411)	0.510	0.245	0.740 (0.718, 0.763)	0.575	0.051
**APO(a) PR**	univariate	0.364 (0.343, 0.385)	0.008	0.284	1.156 (1.126, 1.185)	0.175	**0.021***
	+ age, sex	0.709 (0.690, 0.729)	0.242	**0.034***	1.306 (1.279, 1.332)	0.355	**0.005****
	+ age, sex, SRRE	0.562 (0.538, 0.586)	0.263	0.153	1.161 (1.134, 1.187)	0.417	**0.012***
	+ age, sex, *wIS*	0.824 (0.803, 0.844)	0.254	**0.020***	1.302 (1.275, 1.329)	0.327	**0.006****
	+ age, sex, SRRE, *wIS*	0.787 (0.763, 0.811)	0.336	0.053	1.139 (1.112, 1.166)	0.421	**0.013***
**APO(a) FCR**	univariate	0.443 (0.430, 0.456)	0.143	**0.035***	0.462 (0.442, 0.483)	0.041	0.167
	+ age, sex	0.527 (0.515, 0.538)	0.363	**0.009****	0.424 (0.405, 0.443)	0.191	0.174
	+ age, sex, SRRE	0.587 (0.572, 0.602)	0.312	**0.021***	0.355 (0.334, 0.375)	0.138	0.288
	+ age, sex, *wIS*	0.403 (0.392, 0.414)	0.475	**0.032***	0.443 (0.426, 0.459)	0.423	0.097
	+ age, sex, SRRE, *wIS*	0.396 (0.382, 0.410)	0.471	0.086	0.398 (0.381, 0.414)	0.466	0.144
**Outcome:**		**Predictor: IL-18BP**			**Predictor: hsCRP**		
	**Model**	**B (95% CI)**	**Adj. R^2**	***p*-value**	**B (95% CI)**	**Adj. R^2**	***p*-value**
**Lp(a)**	univariate	0.198 (0.182, 0.214)	-0.015	0.429	0.249 (0.233, 0.264)	0.002	0.316
	+ age, sex	0.246 (0.232, 0.261)	0.141	0.291	0.431 (0.417, 0.445)	0.229	0.068
	+ age, sex, SRRE	0.106 (0.091, 0.121)	0.176	0.665	0.658 (0.638, 0.678)	0.319	0.054
	+ age, sex, *wIS*	0.251 (0.237, 0.264)	0.268	0.246	0.352 (0.339, 0.366)	0.308	0.119
	+ age, sex, SRRE, *wIS*	0.020 (0.008, 0.033)	0.472	0.918	0.356 (0.337, 0.374)	0.512	0.236
**APO(a) PR**	univariate	0.120 (0.104, 0.136)	-0.033	0.635	0.297 (0.282, 0.312)	0.021	0.231
	+ age, sex	0.150 (0.135, 0.165)	0.073	0.534	0.386 (0.371, 0.400)	0.164	0.115
	+ age, sex, SRRE	0.010 (-0.005, 0.026)	0.178	0.966	0.247 (0.225, 0.269)	0.199	0.488
	+ age, sex, *wIS*	0.151 (0.136, 0.166)	0.034	0.540	0.383 (0.367, 0.398)	0.122	0.134
	+ age, sex, SRRE, *wIS*	-0.021 (-0.037, -0.006)	0.178	0.931	0.138 (0.114, 0.162)	0.184	0.719

Outcome:		Predictor: IL-18BP			Predictor: hsCRP		
	**Model**	**B (95% CI)**	**Adj. R^2**	***p*-value**	**B (95% CI)**	**Adj. R^2**	***p*-value**
**APO(a) FCR**	univariate	-0.079 (-0.089, -0.069)	-0.032	0.625	0.048 (0.038, 0.058)	-0.039	0.765
	+ age, sex	-0.097 (-0.106, -0.088)	0.132	0.517	-0.046 (-0.055, -0.036)	0.118	0.771
	+ age, sex, SRRE	-0.097 (-0.107, -0.087)	0.101	0.558	-0.411 (-0.425, -0.397)	0.226	0.078
	+ age, sex, *wIS*	-0.101 (-0.109, -0.093)	0.356	0.437	0.031 (0.022, 0.039)	0.337	0.825
	+ age, sex, SRRE, *wIS*	-0.043 (-0.051, -0.034)	0.378	0.758	-0.217 (-0.230, -0.204)	0.411	0.307

Asterisks (*) indicate *p*-values, $*p<0.05$, $**p<0.01$.

Table S8. Relationships of plasma IL-6, IL-18, IL-18BP, and hsCRP with APO(a) PR or APO(a) FCR per SRRE category.

		Black		Hispanic		White	
**Outcome:**		**Predictor: IL-6**		**Predictor: IL-6**		**Predictor: IL-6**	
	**Model**	**B (95% CI)**	***p*-value**	**B (95% CI)**	***p*-value**	**B (95% CI)**	***p*-value**
**APO(a) PR**	univariate	-0.167 (-0.202, -0.132)	0.765	0.917 (0.864, 0.971)	0.301	0.292 (0.243, 0.340)	0.709
	+ age, sex	-0.380 (-0.440, -0.319)	0.697	1.243 (1.186, 1.300)	0.209	0.855 (0.820, 0.890)	0.228
	+ age, sex, *wIS*	-0.433 (-0.507, -0.359)	0.715	1.485 (1.424, 1.546)	0.180	1.128 (1.117, 1.139)	0.077
**APO(a) FCR**	univariate	-0.229 (-0.257, -0.201)	0.612	0.667 (0.653, 0.702)	0.115	0.622 (0.604, 0.640)	0.079
	+ age, sex	0.273 (0.228, 0.318)	0.705	0.733 (0.712, 0.753)	0.066	0.560 (0.542, 0.579)	0.162
	+ age, sex, *wIS*	-0.349 (-0.382, -0.315)	0.527	0.594 (0.576, 0.612)	0.095	0.552 (0.521, 0.583)	0.393
		**Predictor: IL-18**		**Predictor: IL-18**		**Predictor: IL-18**	
	**Model**	**B (95% CI)**	***p*-value**	**B (95% CI)**	***p*-value**	**B (95% CI)**	***p*-value**
**APO(a) PR**	univariate	0.951 (0.917, 0.983)	0.098	1.496 (1.435, 1.558)	0.158	0.556 (0.419, 0.693)	0.799
	+ age, sex	1.109 (1.078, 1.139)	0.057	1.454 (1.391, 1.516)	0.187	1.806 (1.675, 1.938)	0.433
	+ age, sex, *wIS*	1.216 (1.182, 1.250)	0.063	1.412 (1.344, 1.487)	0.256	2.298 (2.008, 2.588)	0.645
**APO(a) FCR**	univariate	0.428 (0.398, 0.459)	0.390	0.031 (-0.006, 0.067)	0.958	0.132 (0.056, 0.207)	0.913
	+ age, sex	0.626 (0.599, 0.653)	0.179	0.088 (0.055, 0.121)	0.869	0.759 (0.671, 0.848)	0.605
	+ age, sex, *wIS*	0.400 (0.380, 0.420)	0.243	0.204 (0.176, 0.233)	0.657	2.488 (2.432, 2.543)	0.175
		**Predictor: IL-18BP**		**Predictor: IL-18BP**		**Predictor: IL-18BP**	
	**Model**	**B (95% CI)**	***p*-value**	**B (95% CI)**	***p*-value**	**B (95% CI)**	***p*-value**
**APO(a) PR**	univariate	-0.506 (-0.533, -0.479)	0.253	0.159 (0.132, 0.186)	0.712	0.136 (0.093, 0.179)	0.842
	+ age, sex	-0.435 (-0.464, -0.407)	0.363	0.021 (-0.010, 0.052)	0.966	0.371 (0.329, 0.412)	0.591
	+ age, sex, *wIS*	-0.443 (-0.475, -0.411)	0.408	-0.266 (-0.316, -0.216)	0.742	0.875 (0.822, 0.928)	0.417
**APO(a) FCR**	univariate	-0.186 (-0.209, -0.163)	0.618	-0.284 (-0.296, -0.272)	0.165	0.275 (0.252, 0.296)	0.450
	+ age, sex	-0.142 (-0.165, -0.199)	0.698	-0.227 (-0.239, -0.216)	0.259	0.186 (0.160, 0.212)	0.660
	+ age, sex, *wIS*	-0.262 (-0.276, -0.247)	0.288	-0.015 (-0.033, 0.002)	0.956	0.117 (0.066, 0.167)	0.886
		**Predictor: hsCRP**		**Predictor: hsCRP**		**Predictor: hsCRP**	
	**Model**	**B (95% CI)**	***p*-value**	**B (95% CI)**	***p*-value**	**B (95% CI)**	***p*-value**
**APO(a) PR**	univariate	-0.231 (-0.261, -0.201)	0.633	0.856 (0.819, 0.893)	0.179	-0.026 (-0.121, 0.069)	0.986
	+ age, sex	0.054 (0.016, 0.092)	0.929	0.599 (0.552, 0.647)	0.422	-0.472 (-0.605, -0.341)	0.824
	+ age, sex, *wIS*	0.048 (0.005, 0.091)	0.944	0.562 (0.508, 0.616)	0.526	-1.786 (-2.016, -1.557)	0.650

		Predictor: hsCRP		Predictor: hsCRP		Predictor: hsCRP	
	**Model**	**B (95% CI)**	***p*-value**	**B (95% CI)**	***p*-value**	**B (95% CI)**	***p*-value**
**APO(a) FCR**	univariate	-0.570 (-0.591, -0.549)	0.120	-0.266 (-0.287, -0.246)	0.433	-0.801 (-0.846, -0.56)	0.299
	+ age, sex	-0.347 (-0.374, -0.320)	0.427	0.000 (-0.023, 0.021)	0.998	-0.148 (-0.229, -0.067)	0.909
	+ age, sex, *wIS*	-0.183 (-0.202, -0.164)	0.563	0.090 (0.071, 0.109)	0.772	0.381 (0.223, 0.538)	0.880

Black, n = 10; Hispanic, n = 9; White, n = 6.

Table S9. Relationships of plasma IL-6 with APO(a) PR, APO(a) FCR, or plasma Lp(a) levels in Hispanic and White participants combined.

		Hispanic + White		
**Outcome:**		**Predictor: IL-6**		
	**Model**	**B (95% CI)**	**Adj. R^2**	***p*-value**
**APO(a) PR**	univariate	0.749 (0.722, 0.776)	0.129	0.103
	+ age, sex	1.196 (1.171, 1.220)	0.463	**0.010****
	+ age, sex, SRRE	1.004 (0.977, 1.032)	0.460	**0.043***
	+ age, sex, *wIS*	1.337 (1.314, 1.360)	0.549	**0.004****
	+ age, sex, SRRE, *wIS*	1.155 (1.129, 1.181)	0.547	**0.019***
**APO(a) FCR**	univariate	0.741 (0.729, 0.752)	0.541	**0.001*****
	+ age, sex	0.526 (0.516, 0.536)	0.727	**0.006****
	+ age, sex, SRRE	0.610 (0.599, 0.621)	0.739	**0.005****
	+ age, sex, *wIS*	0.486 (0.476, 0.496)	0.744	**0.011***
	+ age, sex SRRE, *wIS*	0.568 (0.557, 0.579)	0.746	**0.010****
**Lp(a)**	univariate	0.007 (-0.024, 0.038)	-0.077	0.988
	+ age, sex	0.668 (0.642, 0.694)	0.419	0.126
	+ age, sex, SRRE	0.394 (0.365, 0.422)	0.461	0.389
	+ age, sex, *wIS*	0.848 (0.836, 0.871)	0.584	**0.035***
	+ age, sex SRRE, *wIS*	0.586 (0.562, 0.609)	0.636	0.146

n = 15. Asterisks (*) indicate *p*-values, *p<0.05, **p<0.01, ***p<0.001.

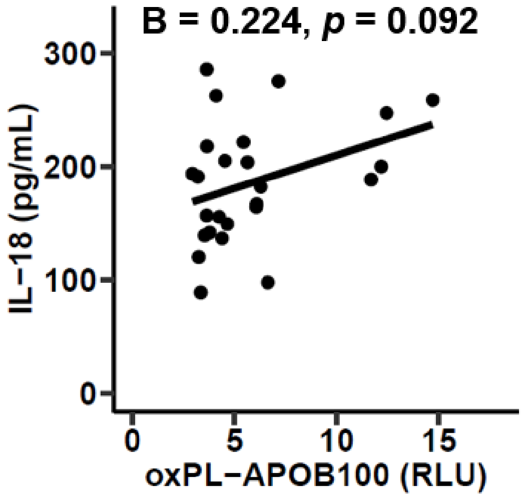

Figure S1. Relationships of oxPL-APOB100 with plasma IL-18 levels. Results are presented as univariate linear regression plots. Relationships of oxPL-APOB100 with plasma IL-18 in all participants (n=25). OxPL, Oxidized phospholipids; IL-18, interleukin-18.

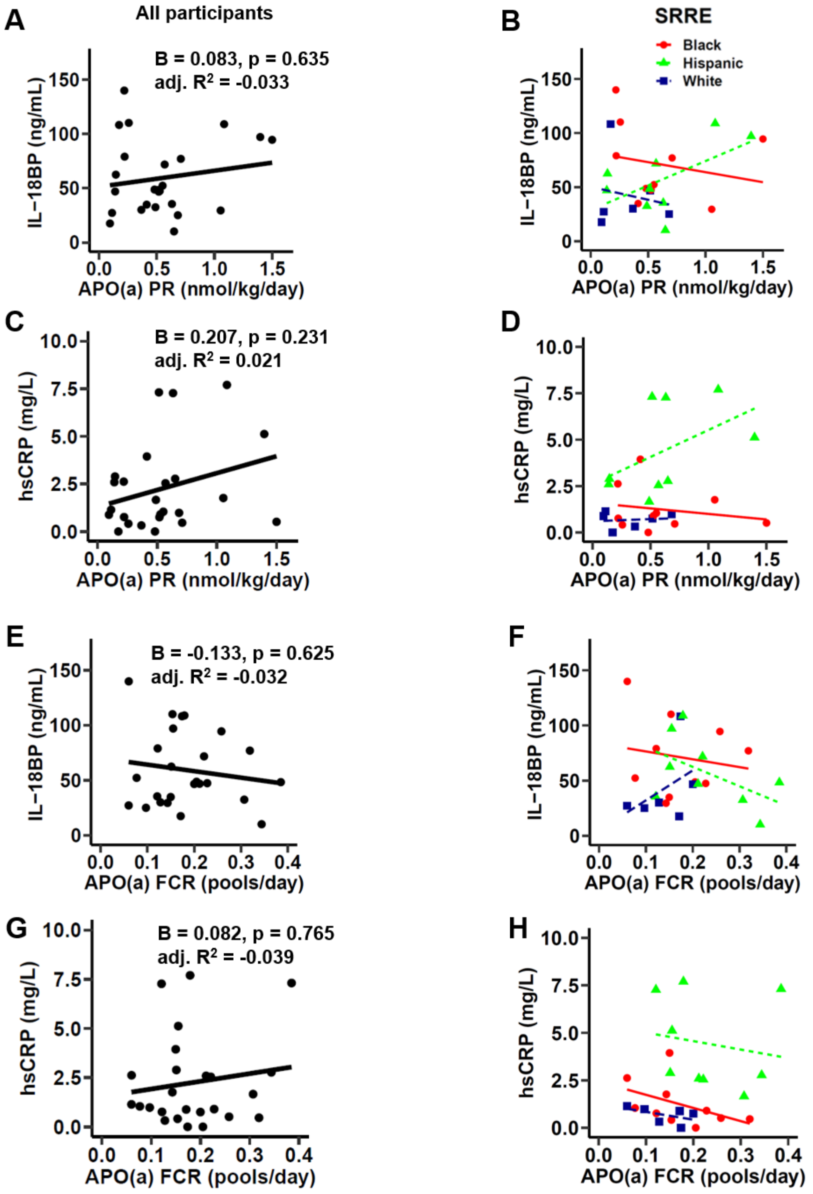

Figure S2. Relationships of APO(a) production rate (PR) or APO(a) fractional catabolic rate (FCR) with plasma IL-18BP or hsCRP levels. Results are presented as univariate linear regression plots. (**A-D**) Relationships of APO(a) PR with IL-18BP (**A**) or hsCRP (**C**) in all participants (n=25), or (**B, D**) separated by three SRRE categories. (**E-H**) Relationships of APO(a) FCR with IL-18BP (**E**) or hsCRP (**G**) in all participants (n=25), or (**F, H**) separated by three SRRE categories. (**A, C, E, G**) B-coefficients (regression coefficients), *p*-values, and adjusted R^2 values are shown. Red dots, Black participants (n=10); green triangles, Hispanic participants (n=9); darkblue squares, White participants (n=6). PR, production rate; FCR, fractional catabolic rate; IL-18BP, interleukin-18 binding protein; hsCRP, high sensitivity C-reactive protein.
